# Effectiveness of Two Endodontic Instruments in Calcium Silicate-Based Sealer Retreatment

**DOI:** 10.3390/bioengineering10030362

**Published:** 2023-03-15

**Authors:** Antoun Farrayeh, Samar Akil, Ammar Eid, Valentina Macaluso, Davide Mancino, Youssef Haïkel, Naji Kharouf

**Affiliations:** 1 Department of Endodontics, Faculty of Dental Medicine, Damascus University, Damascus 0100, Syria; 2 ESTA, School of Business and Technology, 90000 Belfort, France; 3 Pôle de Médecine et Chirurgie Bucco-Dentaire, Hôpital Civil, Hôpitaux Universitaire de Strasbourg, 67000 Strasbourg, France; 4 Department of Biomaterials and Bioengineering, INSERM UMR_S, Strasbourg University, 67000 Strasbourg, France; 5 Department of Endodontics, Faculty of Dental Medicine, Strasbourg University, 67000 Strasbourg, France

**Keywords:** root canal retreatment, calcium silicate-based cement, ProTaper Universal Retreatment, D-Race

## Abstract

The objective of the present in vitro work was to investigate the effectiveness and time required for the removal of calcium silicate-based sealer using two rotary retreatment systems. Sixty extracted, single-canal, lower premolars were used. After obturation using the single-cone technique with calcium silicate-based sealer, samples were divided into four groups according to the technique of desobturation: Group 1 (G1): D-Race; Group 2 (G2): D-Race followed by the use of XP–Endo Finisher R; Group 3 (G3): Protaper Universal Retreatment; and Group 4 (G4): Protaper Universal Retreatment followed by the use of XP–Endo Finisher R. Cone beam computed tomography (CBCT) images were used to calculate the remaining filling materials at the middle and apical thirds. Times required to perform each method were recorded. Scanning electron microscopy (SEM) and digital microscopy were used to evaluate the remaining filling materials. Data were statistically analyzed using the *t*-test and one way ANOVA on ranks tests. No statistically significant difference was found between G1 and G3 after CBCT observations (*p* > 0.05). Xp-Endo Finisher R significantly increased the ability to remove materials regardless of the initially used retreatment system (*p* < 0.05). Statistically significant longer time was found in G3 and G4 compared to G1 and G2, respectively (*p* < 0.05), to reach the full working length. No retreatment system was able to totally remove the calcium silicate-based sealer from the root canal at the middle and apical thirds (*p* > 0.05). Digital microscopy demonstrated that the residual materials were the remaining sealers on the canal walls. SEM showed the mineral depositions of calcium silicate materials onto the canal walls and into the dentinal tubules. However, that calcium silicate materials provide mineral deposition into the dentinal tubules might indicate that the traditional irrigants could not be sufficient to remove calcium silicate-based materials from the root canal, and other agents should be used to make retreatment considerably easier.

## 1. Introduction

Non-surgical endodontic retreatment is the first option after the failure of conventional endodontic treatment [1]. It leads to elimination of the microorganisms that are responsible for persistent infections [2,3]. Bacteria such as *Enterococcus faecalis* remain after primary endodontic treatment in areas that were unreachable by instrumentation and irrigation [4,5]. It is impossible to totally remove the filling material from the root canal during retreatment using conventional instruments and techniques due to their anatomical complexity, especially in oval canals [6,7]. The residual microorganisms and filling materials could affect the final retreatment outcome [8]. In addition, the main purpose of endodontic retreatment is to retrieve healthy periapical tissue. It aims to regain access to the apical region through total removal of filling materials [9].

Removal of gutta-percha can be accomplished using hand files, rotary and reciprocating systems, ultrasonic methods, lasers, and chemical solvents [10,11,12,13,14]. Mechanized protocols were proposed to be supplementary steps after the initial removal of filling materials, including the use of Self-Adjusting File (SAF) instruments (ReDent, Ra’anana, Israel) [15], sonic and ultrasonic tips [7], the XP-endo Finisher (FKG Dentaire, La Chaux-de-Fonds, Switzerland), and the XP-endo Finisher R (FKG Dentaire, La Chaux-de-Fonds, Switzerland), with the main cause of removing the remaining materials from the root canal system.

The use of nickel-titanium (NiTi) rotary retreatment systems is an efficient and safe approach to remove filling materials during root canal retreatment [16]. In addition, several dental companies have introduced NiTi rotary retreatment systems on the dental market. The ProTaper Universal Retreatment system (Dentsply Maillefer, Ballaigues, Switzerland) includes three files—D1, D2 and D3—to remove filling materials from the root canal. These files have a convex triangular cross-section identical to the ProTaper shaping and finishing instruments [17].

The D-RaCe retreatment system (FKG Dentaire, La-Chaux-de Fonds, Switzerland) contains two instruments—DR1 (30/0.10) and DR2 (25/0.04)—for cleaning of the coronal third and to reach the working length (WL), respectively. Both instruments possess an active working tip to promote the initial penetration into the root canal filling material [18].

The XP-endo Finisher R (XPF-R) (FKG Dentaire, Switzerland) is a non-tapered file fabricated with NiTi MaxWire alloy (Martensite-Austenite Electropolish Flex). At temperatures less than 30 °C, this file is straight (martensitic phase “M-phase”), while the placement of this file inside the root canal at body temperature can transform it into an austenitic phase. In this phase, the file takes on a spoon shape in the last 10 mm, with a depth of roughly 1.5 mm. During entry of this file into the root canal, its austenite phase conversion and shape memory enhance its effectiveness in displacing and touching root-obturation materials. Therefore, this profile allows the instrument to attain irregular zones without modifying the original shape of the canal [1,8,19].

Various chemical compositions have been used in endodontic sealers to obturate the root canal with a gutta-percha point. These materials include zinc oxide-eugenol, gutta-percha flow, epoxy-resin, and calcium silicate cements [20]. Calcium silicate (CS) materials, called bioceramics, are considered a breakthrough in endodontic treatment due to their advantageous biocompatibility, antibacterial activity, appropriate filling ability, and good physicochemical properties. Their setting reaction could generate hydroxyapatite on their surfaces and cause tags in the dentinal tubules [21,22]. Different forms of calcium silicate sealers were introduced as powder-liquid and premixed products [23]. Premixed sealers demonstrated greater filling ability and easier application than conventional sealer. Ceraseal (Metabiomed, Korea) is a premixed calcium silicate sealer that demonstrated high obturation quality and appropriate biological, mechanical, and physicochemical properties [23]. The main disadvantage of calcium silicate materials is difficulty with their retreatment, especially at the apical third, and their removal requires both mechanical and chemical procedures [21].

Various dental companies have developed NiTi removal systems such as Mtwo retreatment (VDW, Munich, Germany), R-Endo, and Remover (Micro-Mega, Besançon, France). Hassan et al. studied the retreatment efficiency of R-endo and Xp-endo shaper files in root canals obturated using a bioceramic root canal sealer. The results showed that Xp-endo shaper files were significantly more effective in removing obturation materials. However, neither system totally removed the remaining materials from the coronal, middle, and apical thirds [24]. Donnermeyer et al. studied the retreatability of three calcium silicate sealers and one epoxy resin sealer with four different root canal instruments. They reported that engine-driven NiTi instruments were better suited to remove root canal fillings than stainless steel Hedström files [25].

Until now, there has been no validated protocol for the desobturation of calcium silicate material from the root canal in the literature; thus, research studies are still needed to enhance the efficacy of instruments and solvents in bioceramic retreatment.

The objective of this study was to evaluate the effectiveness of two retreatment instruments with or without a supplementary activation step in the removal of calcium silicate-based sealer. The null hypothesis was that there is no difference between the different instruments and the supplementary cleaning step regarding the quality of calcium silicate retreatment.

## 2. Materials and Methods

### 2.1. Teeth Preparation

After approval by the ethics committee of Damascus University, Damascus, Syria (protocol n. 3215-2020), 63 extracted single canal lower premolars without caries and previous endodontic treatment were selected for the present study. The exclusion criteria were teeth with resorption, incomplete apices, severe curvatures, and cracks. The canal curvature was measured as described in a previous study [26]; thus, teeth with a maximum canal curvature of 15° were selected. Root surfaces were cleaned, and all soft tissue and calculus were removed mechanically with periodontal curettes. Afterwards, the teeth were rinsed and then kept in 0.9% NaCl at 4 °C.

After preparation of the access cavity using diamond burs and ultrasonic tips, a size 10 stainless steel K-file (Mani, Takenzawa, Japan) was inserted to reach the apical foramen. All endodontic steps were performed under 4.5× magnification using Q-Optics Loupes (Q-Optics, Duncanville, TX, USA). To standardize the samples, all tooth crowns were sectioned to reach a root length of 17 mm and a working length (WL) of 16 mm. The same operator prepared all canals using ProTaper Next instruments (Dentsply Sirona, Konstanz, Germany) up to an X2 file at the WL with an electric motor (VDW, Munich, Germany) at a speed of 300 rpm and 3 Ncm of torque. After the use of each instrument, the canals were irrigated with 1 mL of 2.5% NaOCl using a 30-gauge needle. After shaping procedures, the smear layer was eliminated using 5 mL of 17% EDTA and 5 mL of 2.5% NaOCl for final irrigation, and then paper points were used to dry the canal. The single-cone technique was used to obturate the root canal with calcium silicate-based sealer (Ceraseal, Metabiomed, Cheongju, Republic of Korea). The intracanal tip was inserted into the coronal third of the canal, and the sealer was dispensed into the canal. The gutta-percha cone was then painted with the calcium silicate material and slowly inserted into the canal to the appropriate length. Finally, to remove the extra gutta-percha, a hot plugger was used to remove it. The access cavities were filled with a temporary filling material (MD-Temp, Metabiomed, Republic of Korea). The samples were then stored in phosphate-buffered saline (PBS10×, Dominique Dutscher, Bernolsheim, France) for 28 days to insure an appropriate setting of the filling material.

### 2.2. Non-surgical Root Canal Retreatment

**Figure 1:**
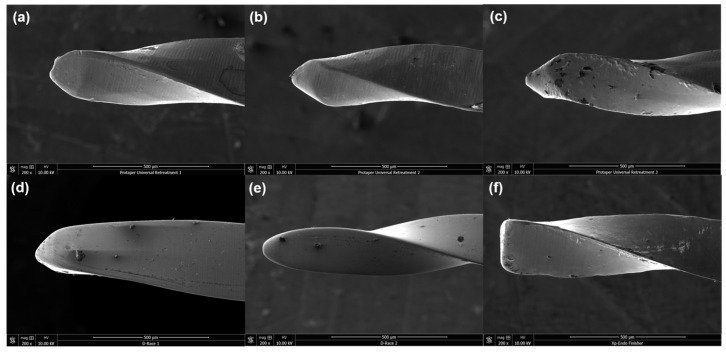
SEM images of the tips of (**a**–**c**) the ProTaper Universal Retreatment D1, D2 and D3, (**d**,**e**) the D-Race DR1 and DR2; and (**f**) the XP–Endo Finisher R file.

After the storage period, coronal access was performed using size 3 and 2 files at 2000 rpm with Peeso drills (Mani, Takenzawa, Japan). The samples were randomly divided into four equal groups (n = 15) as follows:

- Group 1 (G1): D-Race files (Figure 1) were used with a speed of 600 rpm and torque of 1 Ncm. The coronal third of the obturation material was applied with a DR1 file (30/0.10) (Figure 1). Then, the DR2 file (25/0.4) was used for the two other thirds (middle and apical) to attain the working length.
- Group 2 (G2): the same procedure as in G1 was used with an additional step. The XP–Endo Finisher R file (XPF-R) (Figure 1) was used as a supplementary file following the initial retreatment procedures. The XPF-R file was used following the manufacturer’s instructions at a torque of 1 Ncm and a speed of 800 rpm. A contra angle handpiece was used with the instrument. The XPF-R file was placed into the canal with no rotation. Subsequently, the instrument was activated for 1 min using slow and gentle 7- to 8-mm lengthwise movements up to the WL in a brushing action against the root canal walls.
- Group 3 (G3): ProTaper Universal Retreatment files (Figure 1) were used at a speed of 300 rpm with 3 Ncm of torque. A ProTaper D1 file (30/0.09) was used to prepare the coronal third of the canal. At the middle and apical thirds, D2 (25/0.08) and D3 (20/0.07) files were used to remove the filling materials.
- Group 4 (G4): the same procedure as in G3 was used with an additional step. The XP–Endo Finisher R file (XPF-E) (Figure 1) was applied as a supplementary file according the retreatment procedures.

During instrumentation procedures, irrigation was performed with 2 mL of 2.5% NaOCl. All procedures were performed in an incubator at 37 °C. All steps were completed by the same operator. All errors during treatment, including instrument fracture, canal ledges, and blockages, were recorded. In case of a fractured instrument, the tooth was discarded and replaced with another one (three teeth were replaced due to procedural errors). Final irrigation was performed using 5 mL of 17% EDTA and 5 mL of 2.5% NaOCl. After the final irrigation step, the canals were dried with paper points. Two procedural times were recorded to obtain the total working time; including the time needed to attain the WL (T1) and the time needed to remove the gutta-percha (T2). The retreatment procedure was considered complete once the instrument flutes or the irrigation solution had no more residue from the calcium silicate-based sealer or gutta percha material [27].

### 2.3. Remaining Filling Materials Observations

#### 2.3.1. Cone Beam Computed Tomography (CBCT) and Micro-computed Tomography (µCt)

CBCT scans were obtained using a PaX-i3D Green unit (Vatech, Hwaseong-si, Republic of Korea). The samples were exposed to 90 kV and 10.2 mA with an FOV of 5 × 5 cm and an isotropic resolution of 0.1 mm, with 12.57 s of exposure time. The artifacts created by the radiopaque root fillings were eliminated using inbuilt software. The images were evaluated in cross-sections plans, and the percentages of remaining materials at the apical and middle thirds were calculated using the inbuilt software. Finally, the CBCT cross-sections were analyzed, and scores were obtained for all CBCT images by two observers.

After the CBCT observations, the most meaningful sample from each group was analyzed using µCT (IRIS, Inviscan, http://www.inviscan.fr/product_iris_pet_ct.html accessed date: 15 December 2022) to show and evaluate the void percentages in them. The acquisition settings were 2000 projection (60 × 60 × 60 µm 3 voxel size) and 80 kVp. The images were visualized and manipulated using the Mimics Innovation Suite, version 24 (Materialise, Louvain, Belgium).

#### 2.3.2. Digital Microscopy

After CBCT observations, the samples were prepared by creating two shallow longitudinal grooves in the buccolingual direction by a diamond bur. The grooves were created following the canal morphology and curvature. These grooves did not enter into the canal space. Each sample was split by a chisel and mallet to investigate the internal walls [28]. The internal walls of the root canal for each sample were investigated using a digital microscope (KEYENCE, Osaka, Japan) at 100× magnification to analyze the nature of the residual filling materials (gutta-percha and/or sealer), which could not be observed through CBCT analysis.

#### 2.3.3. Scanning Electron Microscopy (SEM)

After digital macroscopy observations, the samples were dried in a graded series of ethanol (50, 70, 95, and 100%) for 10 min each. Finally, these samples were mounted on SEM stubs sputter-coated with gold–palladium (20/80) using a Hummer JR sputtering device (Technics, San Jose, California, USA). The samples were observed at different magnifications (100×−20.000×) with a working distance of 10 mm and a 10-kV acceleration voltage of the electrons through a scanning electron microscope (SEM, Quanta 250 FEG scanning electron microscope, FEI Company, Eindhoven, the Netherlands) [29].

### 2.4. Statistical Analysis

Statistical analysis was performed using SPSS software (version 17, SPSS, Chicago, IL, USA). The normality was verified with the Shapiro–Wilk test. However, when the normality test was not passed, an analysis of the variance on ranks, along with a multiple comparison procedure (Tukey’s test), was performed to determine whether significant differences existed in the remaining material values between the different retreatments. The lapse to complete the retreatment of all groups was determined using the *t*-test, and the chi-square test was used to determine whether significant differences existed in the re-establishing of the working length. In all tests, a statistical significance level of α = 0.05 was adopted.

## 3. Results

### 3.1. Ability to Reach WL and Required Time

**Figure 2:**
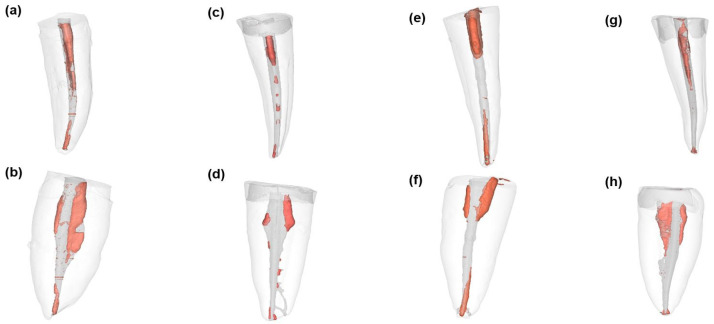
µCt images of the samples after retreatment procedures. (**a**,**b**) G1: D-Race files; (**c**,**d**) G2: D-Race and XP–Endo Finisher R; (**e**,**f**) G3: ProTaper Universal Retreatment; (**g**,**h**) G4: ProTaper Universal Retreatment and XP–Endo Finisher R file.

The WL was re-established in 93.34%, 86.67%, 80%, and 86.67% of teeth for G1, G2, G3, and G4 respectively (*p* > 0.05) (Figure 2). Statistically longer time values were recorded for the groups in which Protaper Universal Retreatment was used compared to the groups in which D-Race was used regardless of the use of XP–Endo Finisher R (Table 1).

### 3.2. CBCT and µCt Evaluations

No significant differences were found for the remaining material percentages between G1 and G3 (*p* > 0.05). The use of XP–Endo Finisher R (G2 and G4) demonstrated statistically greater efficacy in the removal of remaining materials than the other groups (G1 and G3) (*p* < 0.05) (Figure 2 and Table 2).

### 3.3. Digital Microscope Observations

**Figure 3:**
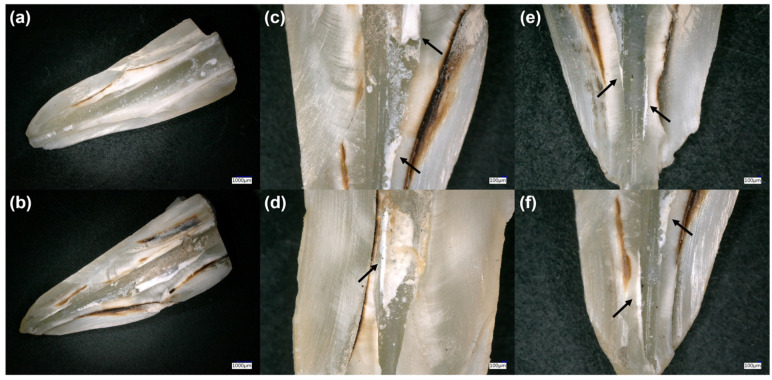
Digital microscope images demonstrate the effectiveness of the retreatment technique and the nature of the residual materials in (**a**,**b**) the root canal at (**c**,**d**) the middle and (**e**,**f**) apical thirds (black arrows).

The use of CBCT did not reveal the nature of the remaining materials; thus, digital microscopy was used to investigate this finding. However, the most common residual materials were the calcium silicate sealers with small amounts of gutta-percha residue, as shown in Figure 3.

### 3.4. SEM Observations

**Figure 4:**
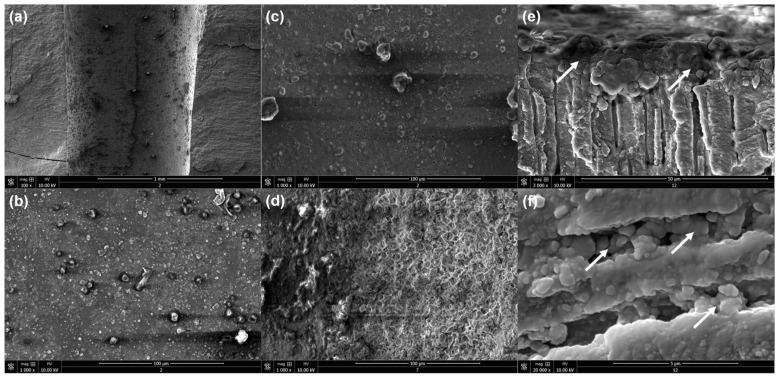
SEM images demonstrate (**a**,**b**) closed dentinal tubules; (**c**,**d**) partially opened dentinal tubules; (**e**) remaining calcium silicate sealer on the root canal walls; (**f**) dentinal tubules filled with mineral components (white arrows).

SEM was used to investigate the quality of smear layer and filling material removal after instrumentation and final irrigation using EDTA and NaOCl. Most dentinal tubules in all of the groups at the middle and apical thirds were not opened after the final irrigation (Figure 4a,b), while only a few zones in G2 and G4 demonstrated partially opened dentinal tubules (Figure 4c,d). Some zones were covered totally with sealer material, and this layer was not eliminated using the different protocols (Figure 4e). This layer covers the entrance of the dentinal tubules (Figure 4e) and prevented complete cleaning of the root canal system. After the immersion period, calcium silicate-based materials ensure the mineral deposition in the dentinal tubules, which could totally close them (Figure 4f). Therefore, the cleaning and opening of the dentinal tubules in root canals could be very difficult after the use of bioceramic material.

## 4. Discussion

Root canal treatments usually fail due to persistent periapical disorders after treatment [30]. Moreover, coronal leakage, periodical caries, necrotic tissue, tooth cracks and fractures, and bacterial biofilms could lead to treatment failure [31]. In addition, these etiological factors should be eliminated to establish adequate periapical recovery; thus, establishing patency and working length in retreatment cases could significantly provide for better periapical healing outcomes [32]. However, retreatment procedures are not always possible due to several factors, including root canal anatomy and resistant filling materials [21,27,33,34].

Currently, calcium silicate materials are used frequently in endodontic practice due to their great biological, mechanical, and physicochemical properties [35,36]. Ceraseal sealer, which was used in the present study, is one of these calcium silicate endodontic products that has demonstrated an alkaline pH and release of Ca^2+^ ions [23]. Therefore, these properties might enhance and provide for the mineralization process and the formation of hydroxyapatite tags in the dentinal tubules [21,23,29,35].

In the present in vitro study, SEM observations, digital analysis, and CBCT investigations demonstrated that there is no retreatment system that could totally eliminate the remaining filling materials and completely open the dentinal tubules. The results demonstrated that there are no statistically significant differences between the two rotary systems (G1 and G3) regarding the efficacy of filling material removal from the root canal at the middle and apical thirds (*p* > 0.05), while the supplementary cleaning step (XPF-R in G2 and G4) enhanced statistically the efficacy of retreatment (*p* < 0.05). These findings suggest that supplementary steps are able to decrease the amount of remaining filling materials. Moreover, the tested techniques in this study showed that they cannot provide for complete removal of the filling materials from the root canal system. Therefore the null hypothesis must be partially rejected.

The findings of the present work demonstrated that retreatment with the D-Race system required less time compared to ProTaper Universal Retreatment files, which might be attributed to the greater efficacy of D-Race files in removing gutta-percha, as well as two instruments being used in the D-Race system compared to three instruments being used in the ProTaper Universal Retreatment. Regarding the amount of remaining filling materials after retreatment, no statistically significant differences were found between the two systems: D-RaCe and ProTaper rotary instruments (*p* > 0.05). In contrast, the addition of a supplementary cleaning step using XPF-R (G2 and G4) demonstrated statistically higher cleaning efficiency results in all root segments compared to the two initial retreatment systems without this supplementary step (*p* < 0.05). This finding could be attributed to XPF-R metallurgy. The manufacture and improvement of these files depend on the shape-memory of its alloy. The file is straight in its M-phase, which is composed in cooled conditions. When the file is subjected to body temperature, its shape transforms into the A-phase. The shape in this phase permits the file to clean the zones that are otherwise inaccessible with regular instruments [37,38]. Therefore, novel instruments, systems, and supplementary steps and techniques are required for remaining filling material removal, especially in oval-shaped root canals, as well as in filled canals with bioceramic materials [28,39,40]. Removal of root filling materials during endodontic retreatment was previously evaluated using stereomicroscopy, scanning electron microscopy, digital microscopy, and x-ray radiography. Some studies used CBCT for the assessment of remaining filling materials. CBCT provides three-dimensional (3D) images on the axial, coronal, and axial planes, and it is capable of visualizing the root canal system and analyzing the quality of cleaning in extracted teeth [10,41].

Digital images demonstrated that most remaining materials in the root canals after retreatment procedures are the residues of calcium silicate sealers, which are stuck on the dentinal walls (Figure 3). Kaloustian et al. [28] used a digital microscope to investigate the amount of the remaining materials after different retreatment methods. Previous studies have reported that gutta-percha material could be eliminated mechanically using instruments and chemically by the application of different solvents [10,11,12,13,14]. In contrast, calcium silicate sealers and cements are very difficult in retreatment due to their high compressive strength, appropriate interactions with dental tissues, and mineralization reactions [20,21,23,29,35,36,42]. A previous study demonstrated that the use of special acids, such as formic acid, is the best route to retreat teeth filled with calcium silicate-based sealer [43].

Various studies have demonstrated that the use of EDTA and NaOCl as irrigants could provide for smear layer removal in permanent and primary teeth [44,45]. In contrast, SEM images in the present study rarely showed dentinal zones with opened dentinal tubules and an eliminated smear layer. Most observations showed debris in the root canal, closed dentinal tubules, remaining calcium silicate particles, and their mineralogical reactions in the dentinal tubules, which are near the principal root canal. In accordance, a previous study demonstrated that the use of calcium silicate-based material by orthograde obturation of the root canals could provide favorable conditions for bacterial entombment by intratubular mineralization [22]. Therefore, plugging the dentinal tubules with calcium silicate materials and/or their mineralogical reactions could close totally the dentinal tubules; thus, special solvent should be used to open them. In contrast, this plugging could enclose the remaining bacteria in the dentinal tubules and kill these microorganisms over time [22].

Finally, the findings of the present study are in accordance with previous studies reporting that retreatment with calcium silicate materials is considered a big challenge in endodontic treatment [21,34,46,47]. These materials could be considered to result in real blockage of the apical foramen, preventing patency of the root canal and the reestablishing of the WL, which are considered to be important outcomes. Further research is needed to evaluate the effects of different acids and solvents accompanying mechanical process and activation in retreatment with calcium silicate materials in straight and curved root canals. Moreover, the mineralization process of the different calcium silicate materials and the time needed to totally close the dentinal tubules will be further investigated. All the samples were observed using CBCT, while fewer samples were analyzed with µCt, which was used to obtain a higher resolution of data that were process using Mimics Innovation Suite software, version 24 (Materialise, Louvain, Belgium). This fact could be considered a limitation of the present work.

## 5. Conclusions

Within the limitations of the present in vitro study, none of the used systems was able to completely remove the CS material from the root canal. No significant difference was found between the two retreatment systems. Supplementary cleaning steps are effective tools to enhance the cleanliness of the root canal after a retreatment procedure. The traditional irrigants (EDTA and NaOCl) could not completely the open dentinal tubules after an obturation procedure using CS materials.

## Figures and Tables

**Table 1:** Time required to re-establish the working length in the different groups. Different superscripted letters (a–d) indicate significant differences between the different groups (*p* < 0.05). Group 1 (G1): D-Race; Group 2 (G2): D-Race followed by the use of XP–Endo Finisher R; Group 3 (G3): Protaper Universal Retreatment; and Group 4 (G4): Protaper Universal Retreatment followed by the use of XP–Endo Finisher R.

	G1	G2	G3	G4	Statistical Analysis (*p* < 0.05)
Time (s)	214 ± 13 ^a^	269 ± 28 ^b^	304 ± 34 ^c^	362 ± 35 ^d^	a < c and b < d

**Table 2:** Residual material percentages after all retreatment techniques. Different superscripted letters (a–d) indicate significant differences between the different groups (*p* < 0.05). Group 1 (G1): D-Race; Group 2 (G2): D-Race followed by the use of XP–Endo Finisher R; Group 3 (G3): Protaper Universal Retreatment; and Group 4 (G4) Protaper Universal Retreatment followed by the use of XP–Endo Finisher R.

	G1	G2	G3	G4	Statistical Analysis (*p* < 0.05)
Middle (%)	12 ± 6 ^a^	8.4 ± 4.1 ^b^	16.4 ± 11.1 ^a^	8.8 ± 3.8 ^b^	b < a
Apical (%)	14 ± 7 ^a^	4.5 ± 0.6 ^b^	15.4 ± 10.6 ^a^	6.5 ± 6.4 ^b^	b < a

## Data Availability

The data presented in this study are available on request from the first author.
